# A Rare Case of Henoch-Schönlein Purpura Following a COVID-19 Vaccine—Case Report

**DOI:** 10.1007/s42399-021-01025-9

**Published:** 2021-09-08

**Authors:** Abdelhamid Naitlho, Wahib Lahlou, Abderrahim Bourial, Hamza Rais, Nabil Ismaili, Imad Abousahfa, Lahcen Belyamani

**Affiliations:** 1 Department of Internal Medicine, Cheikh Khalifa International University Hospital, Mohammed VI University of Health Sciences (UM6SS), Casablanca, Morocco; 2 Cheikh Khalifa International University Hospital, Mohammed VI University of Health Sciences (UM6SS), Casablanca, Morocco; 3 Department of Oncology, Cheikh Khalifa International University Hospital, Mohammed VI University of Health Sciences (UM6SS), Casablanca, Morocco; 4 Department of Emergency, Cheikh Khalifa International University Hospital, Mohammed VI University of Health Sciences (UM6SS), Casablanca, Morocco

**Keywords:** Rheumatoid purpura, Henoch-Schönlein purpura, Anti-SARS-CoV-2 vaccine, COVID-19, Post-vaccinal vasculitis, Vaccine secondary effects

## Abstract

In the COVID-19 pandemic era, anti-SARS-CoV-2 vaccination is considered to be the most efficient way to overtake the COVID-19 scourge. Like all medicines, vaccines are not devoid of risks and can in rare cases cause some various side effects. The objective of this case report is to highlight this unusual presentation of Henoch-Schönlein purpura following an anti-COVID-19 vaccination in a 62-year-old adult. The 62-year-old patient admitted to the emergency room for a petechial purpuric rash, sloping, occurring within hours, involving both legs and ascending. The clinical signs also included polyarthralgia and hematuria. Reported in the history the notion of an anti-COVID-19 vaccination 8 days prior to the onset of symptomatology. In the case of our patient, we retain the diagnosis of rheumatoid purpura based on the EULAR/PRINTO/PReS diagnostic criteria. Corticosteroid therapy (prednisone) was started, resulting to a rapid regression of clinical and laboratory symptoms, few days after the treatment. Patient was asymptomatic on subsequent visits. The low number of published cases of post-vaccine vasculitis does not question the safety of vaccines, but knowledge of such complications deserves to be known in order to avoid new immunizations that could have more serious consequences, and to avoid aggravating or reactivating a pre-existing vasculitis.

## Introduction

Vasculitides are a group of heterogeneous diseases characterized by inflammation of the vascular wall. Their pathophysiological mechanism remains complex and little known. A combined role of a particular genetic background and environmental factors (particular exposures to toxins or infections such as the hepatitis B or C virus) can explain the diseases [1].

Among these factors, we find vaccines. Several cases of post-vaccine vasculitis have been described in the literature.

We report here a case of post-COVID-19 vaccine rheumatoid purpura diagnosed within the internal medicine department, at the Cheikh Khalifa International University Hospital in Casablanca, Morocco.

## Observation

**Fig. 1:**
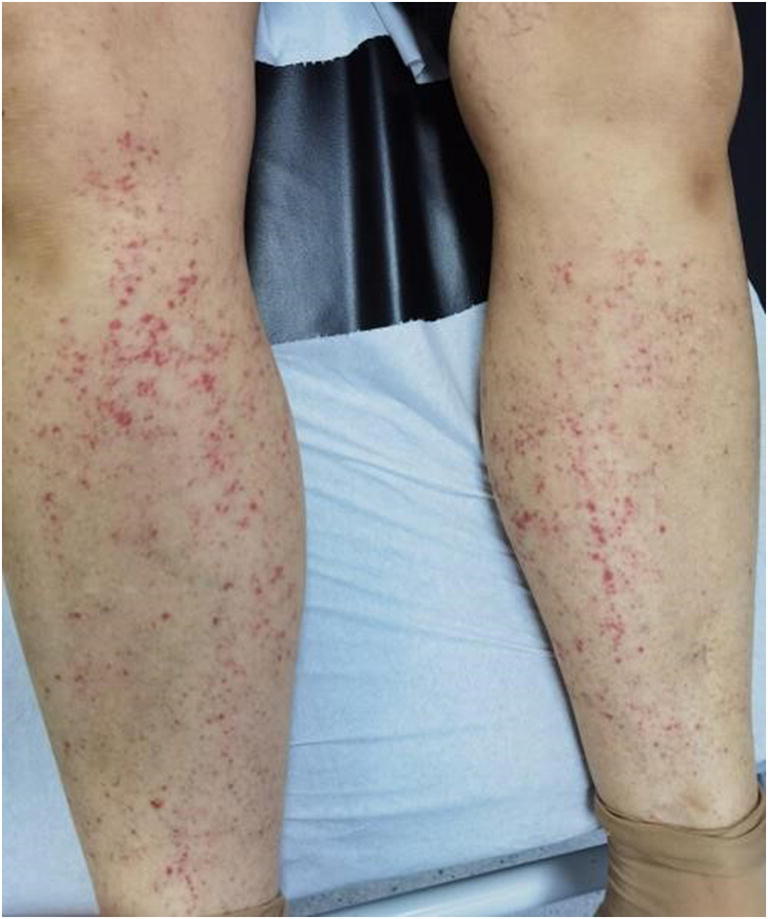
Petechial purpuric rash

This is a 62-year-old patient admitted to the emergency room for a petechial purpuric rash, sloping, occurring within hours, involving both legs and ascending (Fig. 1).

The patient also reports bilateral polyarthralgia of the knees and ankles which had occurred a day before.

His history includes osteosarcoma of the left tibia at the age of 5 treated with surgery and chemotherapy, intercostal shingles at the age of 16, tonsillectomy at the age of 20, and COVID-19 infection in October 2020 without notable complications. We also note in its medical history the notion of first dose anti-COVID-19 vaccination [Oxford-AstraZeneca COVID19 = ChaAdOx1 nCoV-19 vaccine (AZD1222)] 8 days before the onset of its symptomatology.

## Investigations

The biological assessment carried out in the emergency department found an inflammatory syndrome (CRP at 25.64 mg/L) and D-dimers at 1490 ng/mL. In addition, no anomaly was found in the blood count.

An autoimmune assessment was performed in the internal medicine consultation the next day and found positive ANA at 640 and a rheumatoid factor at 215 IU/mL.

A cytobacteriological urine test was performed and showed microscopic hematuria at 2000 RBCs/mL.

**Fig. 2:**
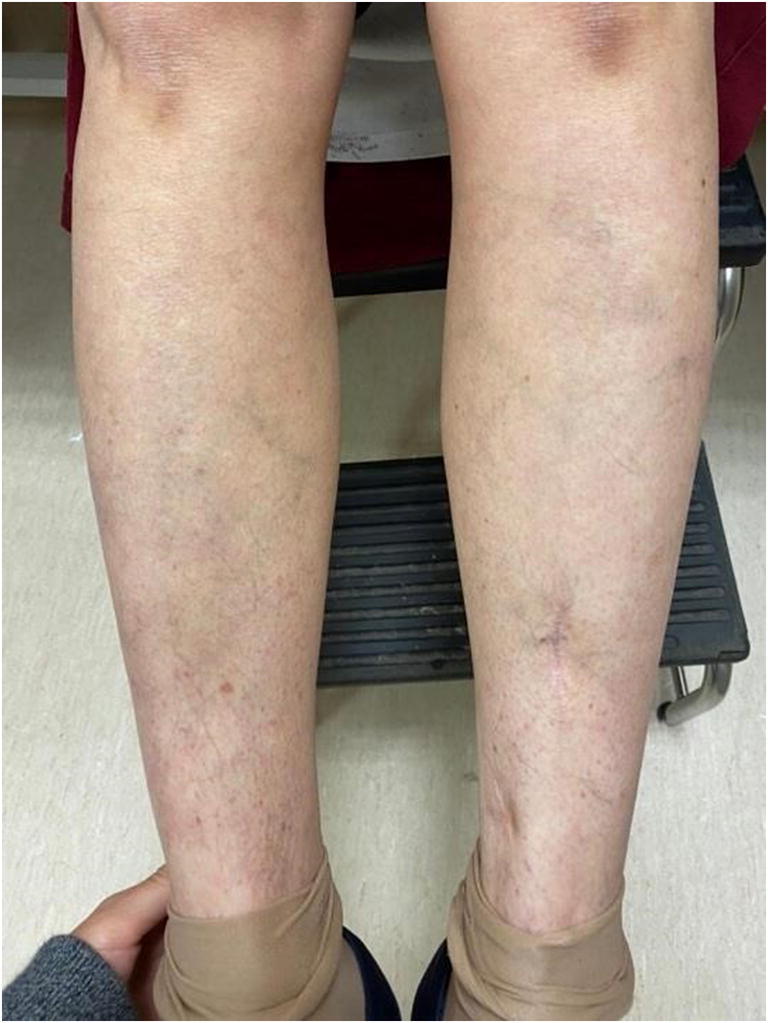
Healing of lesions after 7 days of corticosteroid therapy

Corticosteroid therapy (prednisone) was started at a dose of 40 mg per day for 7 days, leading to a rapid regression of clinical and laboratory symptoms: regression/healing of the lesions (Fig. 2), a negativation of ANA, a decrease in rheumatoid factor to 127 IU/mL, D-dimers to 360 ng/mL and CRP to 4.5 mg/L.

## Discussion

To our knowledge, up to date, this is the second case of post-vaccine vasculitis described in the literature [2], as part of the SARS-CoV-2 vaccine in general, and first AZD1222 (ChAdOx1 nCoV-19) in particular. And the first post-COVID-19 vaccine Henoch-Schönlein vasculitis was described.

Henoch-Schönlein disease, or rheumatoid purpura, is vasculitis affecting capillaries in the blood as a result of IgA deposition in the vessel walls [3, 4].

The clinical signs associated with Henoch-Schönlein disease are a change in general condition and fever, but also other more specific signs [3, 5]:

- **Symmetrical vascular purpura**, not erased by diascopy, most often present in areas of pressure with the primary lesion of petechia or even bruising, or necrosis or hemorrhagic bubbles in adults. The skin lesions disappear in about 15 days.
- **Arthralgia** localized to the knees and ankles in more than ¾ of cases.
- **Diffuse abdominal pain **of significant intensity in 60% of the cases identified.
- Intussusception is possible in children and is most often accompanied by vomiting and upper or lower gastrointestinal bleeding.
- **Glomerular renal impairment** in 40% of cases, which results in proteinuria and/or hematuria, without the presence of renal failure in most cases.

The exact pathophysiological mechanism in post-vaccine vasculitis is still poorly understood. The most commonly discussed hypothesis is immunological, related to the presence of immune complexes starting from the vaccine antigen. The pathophysiology is similar to that linking periarteritis nodosa to HBV Ag-Ac immune complexes. However, in the majority of vasculitis, the causative agent is not known [6].

In 2010, EULAR/PRINTO/PReS defined criteria (Table 1) that have become the benchmark for the diagnosis of Henoch-Schönlein disease (HSP). The diagnosis of HSP is clinical. These criteria, applicable to adults, have a diagnostic sensitivity of 79.2% and a specificity of 86%, which encourages their use for any patient suffering from this pathology [10].

**Table 1:** Diagnostic criteria for Henoch-Schönlein purpura (HSP), as developed by EULAR/PRINTO/PRES [7–9]

**Criterion**	**Description**
**Mandatory criterion**	Purpura or petechiae with lower limb predominance
**Minimum 1 out of 4 criteria**	1- Diffuse abdominal pain with acute onset 2- Histopathology showing leukocytoclastic vasculitis or proliferative glomerulonephritis with predominant immunoglobulin A (IgA) deposits 3- Arthritis or arthralgia of acute onset 4- Renal involvement in the form of proteinuria or hematuria

In the case of our patient, we retain the diagnosis of rheumatoid purpura in front of:

- **Petechial purpuric rash** *(mandatory criterion)***.**
- **Polyarthralgia.**
- **Hematuria** at cytobacteriological urine test.

AZD1222 (ChAdOx1 nCoV-19) is a vaccine against SARS-CoV-2 which uses a non-replicating chimpanzee adenovirus as a vector (ChAdOx1) and is modified to induce the S protein of SARS-CoV-2. AZD1222 was developed by the University of Oxford and AstraZeneca [11].

Globally, as of 5:15 p.m. CEST on May 10, 2021, 157,973,438 confirmed cases of COVID-19, including 3,288,455 deaths, have been reported to the WHO. As of May 10, 2021, a total of 1,206,243,409 doses of vaccine have been administered [12].

In Morocco, as of January 3, 2020, at 3:52 p.m. CET, on May 10, 2021, there have been 513,864 confirmed cases of COVID-19 with 9072 deaths, reported to the WHO. As of May 10, 2021, a total of 9,325,597 doses of vaccine have been administered [13].

Morocco is one of the best-known countries in terms of anti-SARS-CoV-2 vaccine strategy in the world. The vaccination rate in Morocco (9.75%) accomplished in a single month, which slightly surpasses that of France (6.97%), Italy (7.12%), and Spain (7.71%) in 2 months of vaccination [14].

Priority is given to people on the front lines, such as health workers, teachers, security forces, law enforcement officials, elders, and people with underlying health conditions. Thereafter, vaccination will be extended to the general population by age group [14].

All medicines, including vaccines, have side effects that vary in severity (including death). However, despite the risks, it is necessary to be vaccinated given the clearly established benefits from a public health point of view: Vaccines prevent between 2 and 3 million deaths each year from infectious diseases [15].

Given the high incidence of infectious-starting autoimmune diseases in COVID-19 infection (Kawasaki diseases, Guillain-Barre syndromes, autoimmune myasthenia gravis). We tried through this work to propose a research hypothesis which aims to demonstrate a link between the particles of the anti-SARS-CoV-2 vaccine and Henoch-Schönlein vasculitis.

Considering the fact that our patient has a history of recent COVID-19 infection in October 2020, the anti-SARS-CoV-2 vaccine could have induced a “trained immunity” in already vulnerable patients (history of COVID-19), given the fact that the patient has already been sensitized to SARS-CoV-2 components, particularly the spike protein which is used in the COVID-19 vaccines [16].

In the same logic, a lot of infections including COVID-19 are associated with the release of cytokines, the primary immune system regulators. Some of these cytokines can dysregulate the immune system and contribute to an inflammatory state called the cytokine storm [17] that increases between day 5 and day 13 [18]. It is known in the literature that the cytokine storm can be the gateway to certain auto-immune diseases.

A case of a mild itchy erythematous macular morbilliform rash in the lower back 48 h after administration of the first dose of Pfizer-BioNtech COVID-19 mRNA vaccine has been described in the literature. Then, a more significant recurrent morbilliform rash has appeared on both flanks and flesh-colored papules on the right flank with itching and significantly increased bodily surface damage after 48 h of the second booster dose [19].

The association between the anti-SARS-CoV-2 vaccine and the development of HSP is notable. We cannot explain the immunopathogenic link between ChAdOx1 nCoV-19 vaccine and HSP, but we believe that practitioners should be aware of this possible complication and evaluate other cases of post-vaccine vasculitis with keeping causality in mind. However, HSP is rarely used with vaccines and is not expected to affect vaccine use.

## Conclusion

Although the existence of post-vaccine vasculitis has yet to be formally demonstrated, we are adding this case of post-vaccine rheumatoid purpura to the already existing list.

The low number of published cases of post-vaccine vasculitis does not question the safety of vaccines, but knowledge of such complications deserves to be known in order to avoid new immunizations that could have more serious consequences, and to avoid aggravating or reactivating a pre-existing vasculitis.

## Abbreviations

SNFMI: French National Society of Internal Medicine
COVID-19: coronavirus disease 2019
SARS-CoV-2: severe acute respiratory syndrome coronavirus 2
CRP: C-reactive protein
ANA: anti-nuclear antibodies
IgA: immunoglobulin A
Ag-Ac: antigen-antibody
HBV: hepatitis B virus
EULAR: European League Against Rheumatism
PRINTO: The Pediatric Rheumatology INternational Trials Organization
PReS: Pediatric Rheumatology European Society
HSP: Henoch-Schönlein purpura
CET: Central Europe Time
WHO: World Health Organization
mRNA: messenger ribonucleic acid
